# Special Nuclear Structures in the Germinal Vesicle of the Common Frog with Emphasis on the So-Called Karyosphere Capsule

**DOI:** 10.3390/jdb11040044

**Published:** 2023-12-12

**Authors:** Dmitry S. Bogolyubov, Sergey V. Shabelnikov, Alexandra O. Travina, Maksim I. Sulatsky, Irina O. Bogolyubova

**Affiliations:** Institute of Cytology of the Russian Academy of Sciences, St. Petersburg 194064, Russia; buddasvami@gmail.com (S.V.S.); alotra1234@gmail.com (A.O.T.); m_sulatsky@mail.ru (M.I.S.); ibogol@mail.ru (I.O.B.)

**Keywords:** germinal vesicle, nuclear compartments, karyosome, karyosphere, karyosphere capsule, electron microscopy, nuclear actin, *Rana temporaria*

## Abstract

The karyosphere (karyosome) is a structure that forms in the oocyte nucleus—germinal vesicle (GV)—at the diplotene stage of meiotic prophase due to the assembly of all chromosomes in a limited portion of the GV. In some organisms, the karyosphere has an extrachromosomal external capsule, the marker protein of which is nuclear F-actin. Despite many years of theories about the formation of the karyosphere capsule (KC) in the GV of the common frog *Rana temporaria*, we present data that cast doubt on its existence, at least in this species. Specific extrachromosomal strands, which had been considered the main elements of the frog’s KC, do not form a continuous layer around the karyosphere and, according to immunogold labeling, do not contain structural proteins, such as actin and lamin B. At the same time, F-actin is indeed noticeably concentrated around the karyosphere, creating the illusion of a capsule at the light microscopy/fluorescence level. The barrier-to-autointegration factor (BAF) and one of its functional partners—LEMD2, an inner nuclear membrane protein—are not localized in the strands, suggesting that the strands are not functional counterparts of the nuclear envelope. The presence of characteristic strands in the GV of *R. temporaria* late oocytes may reflect an excess of SMC1 involved in the structural maintenance of diplotene oocyte chromosomes at the karyosphere stage, since SMC1 has been shown to be the most abundant protein in the strands. Other characteristic microstructures—the so-called *annuli*, very similar in ultrastructure to the nuclear pore complexes—do not contain nucleoporins Nup35 and Nup93, and, therefore, they cannot be considered autonomous pore complexes, as previously thought. Taken together, our data indicate that traditional ideas about the existence of the *R. temporaria* KC as a special structural compartment of the GV are to be revisited.

## 1. Introduction

The key structures that create the specific compartmentalization of any eukaryotic cell are the nuclear envelope (NE) and the nuclear lamina—a dense network of intermediate filaments, particularly lamins, and a complex set of lamin-associated transmembrane proteins [1]. Simultaneous interactions of the barrier-to-autointegration nuclear factor (BAF/BANF1) with chromatin and inner nuclear membrane proteins belonging to the LEM-D (the lamina-associated polypeptide 2, Emerin, MAN1 domain) family ensures the association of chromosomes with the NE and makes a significant contribution to multiple aspects of genome maintenance [2,3], including in germ cells [4].

Compared to somatic cells, growing female germ cells—the oocytes—are unusual highly specialized cells that are in the process of division, namely at the diplotene stage of meiotic prophase, i.e., during the period of the oocytes’ large growth. Regardless of the specifics and types of female meiosis and oogenesis, the beginning of oocyte growth is accompanied by the separation of chromosomes from the NE. Both the NE and the nuclear lamina are involved in this process, which is supported by special molecular mechanisms which ensure the correct segregation of oocyte chromosomes and the further formation of functional gametes [5]. In developing oocytes of *Drosophila melanogaster*, chromosomes detach from the NE due to the phosphorylation of the BAF by the conserved nucleosome histone kinase NHK-1 (*Drosophila* Vrk-1), resulting in the formation of a heterochromatin structure called the karyosome [6].

The karyosome—also termed the karyosphere [7,8]—is a meiosis-specific and evolutionarily conserved structure. It appears at the prolonged diplotene stage of meiotic prophase in many (but not all) animals due to the assembly of all chromosomes together in a more or less compact body that occupies a very limited area of the giant oocyte nucleus, called the germinal vesicle (GV) at this stage. The molecular mechanisms of karyosome/karyosphere formation [6,9,10,11], as well as the involved genes [12], have been studied in detail for *Drosophila* oogenesis. However, the biological significance of this peculiar superstructural nuclear compartment is not fully understood, even though more than 120 years have passed since the discovery of the karyosphere [13]. One hypothesis is that karyosphere formation contributes to the proper assembly of the spindle and the fidelity of the first meiotic division during the development of large oocytes [14].

The karyosphere can exhibit amazing morphological diversity even among closely related invertebrate and vertebrate species. At a very first approximation, several plans for its structure can be distinguished [8], although this nomenclature is quite formal: (i) a simple tangle of chromosomes, or the karyosome (like in *Drosophila*); (ii) a tangle of chromosomes associated with a fibrous F-actin-containing “shell” that is located on the outside of the chromosomes and is, therefore, called the karyosphere capsule (KC)—e.g., in some insects and also believed to exist in the frog *Rana temporaria*; and (iii) the so-called inverted karyosphere, when chromosomes are joined together on the surface of an extrachromosomal central body. The last type of karyosphere is characteristic, in particular, of some mammals, including mice and humans. In this case, the chromosomes are assembled around a special nuclear organelle called the nucleolus-like body, or atypical nucleolus [15,16,17]. No KC is formed in this case.

In some organisms, as mentioned above, the karyosphere can be additionally separated from the rest of the nucleoplasm by a fibrous superstructural formation, called the KC, a special extrachromosomal compartment within the GV [7,8]. German zoologist Wagner was the first to mention the development of the KC in the GV of the common frog *R. temporaria*, and this was exactly 100 years ago [18]. The KC concept has been further developed through the use of electron microscopy to examine the GVs of the common frog [19] and also insects—e.g., the pearly green lacewing *Chrysopa perla* [20]. An early electron microscopic study performed on the GV of *R. temporaria* late vitellogenic oocytes revealed specific non-membranous strands that connect distinctive nucleoplasmic microstructures called *annuli* [19]. Intriguingly, these *annuli* had a strict morphological similarity to nuclear pore complexes and, therefore, were called autonomous pore complexes. The authors believed that they were seeing elements of the “Wagner capsule” at the ultrastructural level, which consists of the aforementioned strands.

**Figure 1:**
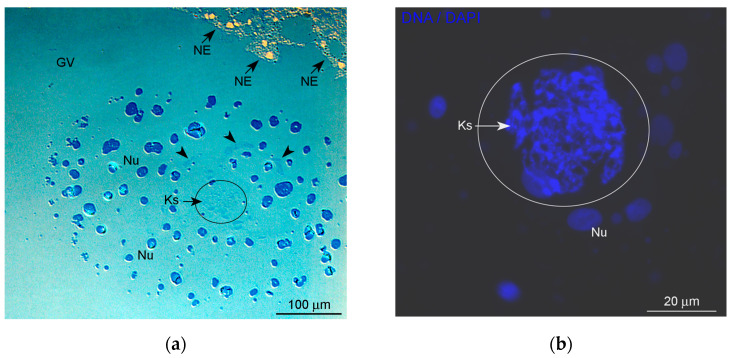
General morphology of the karyosphere-containing region of the *Rana temporaria* germinal vesicle in late vitellogenic oocytes: (**a**) semithin section across a noticeable nucleolar assemblage with the karyosphere inside, after methylene blue staining; and (**b**) the karyosphere/karyosome—a tangle of condensed chromosomes—after DAPI staining. GV, nucleoplasm of the oocyte nucleus (germinal vesicle); Ks, karyosphere, the GV region containing the karyosphere is additionally encircled; NE, nuclear envelope; Nu, amplified nucleoli; arrowheads indicate a fibrous extrachromosomal material in the vicinity of the karyosphere.

The idea of the structural stability of the *R. temporaria* KC [7] is reinforced by the fact that, in various amphibians, both anurans and caudates, the chromosomes of late oocytes, gathered into a karyosphere, are enclosed in a large assemblage of extrachromosomal nucleoli (Figure 1a), which has been well known since the second half of the 19th century [21]. This “nucleolar cloud” occupies the central part of the GV, consists of hundreds of amplified nucleoli [22], and can be easily isolated manually along with the chromosomes. This complex entirely maintains its entire stability for a certain time [19]. The nucleolar conglomerate (cloud, assemblage) and the karyosphere are two distinct nuclear compartments. Despite their close proximity, the amplified nucleoli are not part of the karyosphere itself, which, by definition, is a chromatin compartment. Likewise, this nucleolar assemblage obviously cannot be considered as a capsule, which, by definition, is a filamentous structure [8].

Unlike a somatic cell, amphibian GV contains a large amount of nuclear actin, predominantly in a polymerized form (F-actin), which can help maintain the stability of GV structures [23]. The presence of an extensive network of actin filaments was documented for the *R. temporaria* GV in an early immunogold labeling electron microscopic study [24], although neither the karyosphere nor its capsule were discussed there. Considering that nuclear actin is the main structural protein of the KC in other organisms—namely, in some insects [25,26,27]—F-actin is considered to be a signature component of the KC in a general sense, including in the common frog [8]. Another prediction was made, according to which nucleoplasmic lamins could participate in KC formation, and also some nucleoplasmic nucleoporins were revealed in the karyosphere area of the *R. temporaria* GV [28], although the precise ultrastructural localization of these NE-related proteins has not been documented yet.

Taking into account the long-history of the KC concept, we performed a special study to verify our hypothesis that the *R. temporaria* KC is a special structural compartment of the GV, which may demonstrate some reminiscence of the insect KC and/or the NE, and is involved in the regulation of the spatial organization of diplotene bivalents, gathering into the karyosphere after the natural detachment of oocyte chromosomes from the NE. We intended to pay special attention to both nuclear actin and the role of NE proteins in the interactions of chromatin with extrachromosomal structures of the *R. temporaria* GV. Despite the non-membrane nature of the KC, we expected to find some proteins of the NE—e.g., lamins, nucleoporins, and LEMD-proteins—which could interact with chromosomes via BAF to maintain the integrity of the GV structure.

Surprisingly, we found that F-actin was indeed noticeably concentrated around the *R. temporaria* karyosphere but did not accumulate in the aforementioned extrachromosomal strands. Although lamins, nucleoporins, LEMD-proteins, and BAF were indeed located in the karyosphere-containing region of the GV, they were also not concentrated in the strand. Therefore, in our opinion, the existence of *R. temporaria* KC in its traditional sense is apparently somewhat exaggerated.

## 2. Materials and Methods

### 2.1. Animals and Oocyte Retrieval

Sexually mature females of the European common frog *Rana temporaria* L.—also known as the grass frog—were collected from its natural habitat in the vicinity of St. Petersburg either in October (before the onset of hibernation) or at the end of April (just before the start of the breeding season). The frogs were kept in a refrigerator at 4 °C and washed twice a week with cold water. Our work with these animals complied with all ethical rules (3R principles). The number of animals/oocytes used in the study is presented in Supplementary Table S1.

The latest vitellogenic oocytes were used, corresponding to stage VI [29] or a slightly later stage, judging by the picture given in another paper [18]—i.e., ready for ovulation but still containing an intact GV with a fully developed karyosphere. Spring frogs naturally contain oocytes of this stage [19]. To obtain oocytes of the appropriate stage from autumn frogs, the animals were stimulated with a double injection of 500 IU of hCG (Chorulon, Intervet, Boxmeer, The Netherlands) dissolved in 1 mL of 0.65% NaCl, with an interval of 24 h between injections [30].

The animals were sacrified by decapitation with immediate subsequent spinal cord destruction and obligatory control of spinal reflex disappearance, which complies with the international principles for the humane treatment of laboratory animals. Ovary fragments and separate oocytes were isolated in an OR2 medium [31], containing 82.5 mM of NaCl, 2.5 mM of KCl, 1.0 mM of CaCl_2_, 1.0 mM of MgCl_2_, 1.0 mM of Na_2_HPO_4_, and 5.0 mM of HEPES, with a pH of ~7.8.

### 2.2. Primary Antibodies

The following primary antibodies—monoclonal (mAbs) and polyclonal (pAbs)—were used: mouse mAb against double stranded (ds) DNA (MAB 030, Hemicon, Temecula, CA, USA); rabbit pAb against the N-terminus of actin (Sigma-Aldrich, A2103); goat pAb against lamin B (sc-6217, Santa Cruz Biotechnology, Dallas, TX, USA); Nup35 goat pAb (sc-74762, Santa Cruz Biotechnology, Dallas, TX, USA), Nup93 mouse mAb (sc-374399, Santa Cruz Biotechnology, Dallas, TX, USA); rabbit pAb to BANF1/BAF (ab231331, Abcam, Cambridge, UK); BANF1/BAF mouse mAb (ab248281, Abcam, Cambridge, UK); LEMD2 rabbit pAb (PA5-53589, Invitrogen, Thermo Fisher Scientific, Waltham, MA, USA); mouse mAb to SMC1L1 (WH000824M1, Sigma-Aldrich, St. Louis, MO, USA); and rabbit pAb to SMC1A (ab21583, Abcam, Cambridge, UK).

### 2.3. Western Blot Analysis

Whole oocytes were dissolved in a Laemmli sample buffer. MHeLa cells were cultured in a Dulbecco’s modified Eagle low-glucose medium (DMEM LG, Gibco, Gaithers, MD, USA) supplemented with 10% FBS (fetal bovine serum; HyClone, Salt Lake City, UT, USA), 100 U/mL of penicillin, and 100 µg/mL of streptomycin (Gibco) at 37 °C with 5% CO_2_ in a humidified incubator. The cells were lysed in a Laemmli sample buffer and whole cell proteins were used as a positive control. The protein samples and the pre-stained protein molecular weight (M_w_) marker (#26616, Thermo Scientific, Waltham, MA, USA) were separated using SDS-PAGE [32] and then transferred to a PVDF membrane (Thermo Fisher Scientific). After blocking in 5% milk, the membranes were incubated at 4 °C overnight with primary antibodies against the following proteins, as indicated above: actin (diluted 1:4000); lamin B (diluted 1:2000); Nup35 (diluted 1:2000); Nup93 (diluted 1:2000); BANF1/BAF (diluted 1:2000); LEMD2 (diluted 1:2000); SMC1A (diluted 1:2000); and SMC1L1 (diluted 1:2000). After washing, the membranes were incubated at room temperature for 1.5 h with alkaline phosphatase (AP)-conjugated goat anti-rabbit IgG (diluted 1:10,000; NB730-AP, Novus Biologicals, Littleton, CO, USA), goat anti-mouse IgG (diluted 1:10,000; A3562, Sigma, Burlington, MA, USA), or donkey anti-goat IgG (diluted 1:10,000; A16002, Invitrogen Carlsbad, CA, USA) secondary antibodies. After washing, the AP activity was visualized using a freshly prepared 0.02% BCIP and 0.03% NBT in a solution of 50 mM of Tris-HCl with a pH of 9.5, 5 mM of MgCl_2_, and 100 mM of NaCl.

### 2.4. Fluorescent Microscopy

Oocyte nuclei (GVs) were manually isolated from vitellogenic oocytes in a “5:1 + PO_4_” solution [33] containing 17.0 mM of NaCl, 83.0 mM of KCl, 6.5 mM of Na_2_HPO_4_, 3.5 mM of KH_2_PO_4_, 1.0 mM of MgCl_2_, and 1.0 mM of dithiothreitol (DTT), with a pH of 7.2. The isolated GVs were fixed for 60 min at room temperature in a 4% formaldehyde solution containing 0.5% Triton X-100 in PBS and then overnight in 2% formaldehyde in PBS at 4 °C.

To reveal the F-actin, the GVs were stained with 2 μg/mL of phalloidin–rhodamine for 60 min in a moist chamber at room temperature, placing pieces of hair under a cover glass to avoid too much squashing. Before immunostaining with primary antibodies, the GVs were incubated in 10% fetal serum (Gibco Laboratories, Grand Island, NY, USA) for 10 min at room temperature to prevent non-specific antibody binding. Antibody staining was performed overnight in a moist chamber at 4 °C. After rinsing in PBS, the GVs were placed in a solution of secondary antibodies conjugated with FITC or with desired wave-length Alexa fluorochromes for 90 min at room temperature and mounted in a Vectashield medium (Vector Laboratories, Burlingame, CA, USA) containing 1 μg/mL of DAPI for DNA (karyosphere) detection. The preparations were examined in a Leica TCS SP5 confocal microscope equipped with a set of appropriate lasers and a 40×/1.25 objective. Localization analysis was performed using ImageJ 1.48v and the RGB Profile Plot plugin.

### 2.5. Conventional Electron Microscopy

For the conventional transmission electron microscopy, the oocytes were fixed in 2.5% gluraldehyde in 0.05 M of cacodylate buffer, with a pH of 7.3, for 1.5 h and then in 2% OsO_4_ in the same buffer for 1 h. After dehydration in an ascending series of ethanol, the specimens were embedded in a Spurr low-viscosity resin (Electron Microscopy Sciences, Hatfield, PA, USA) according to the manufacturer’s recommendations. Before cutting ultrathin sections, semithin sections, 0.5 μm in thickness, were prepared for orientation in the material and selection of the place of interest, which were stained with 1% methylene blue in 1% borax. The ultrathin sections collected on nickel grids were contrasted using uranyl acetate and lead citrate and examined in a Libra 120 transmission electron microscope (Carl Zeiss, Oberkochen, Germany) at 80 kV.

### 2.6. Immunoelectron Microscopy

Whole oocytes were prefixed for 2 h in 4% formaldehyde freshly prepared from paraformaldehyde and 0.5% glutaraldehyde in PBS, then postfixed overnight in 2% formaldehyde in PBS at 4 °C. After rinsing in PBS containing 5 mM of NH_4_Cl and subsequent dehydration in an ethanol series, the oocytes were embedded in LR white resin of medium grade (Sigma). The ultrathin sections were incubated for 10 min in a blocking buffer containing 0.5% fish gelatin (Sigma) and 0.02% Tween-20 in PBS, with a pH of 7.4. After blocking, the grids with the sections were incubated in a primary antibody solution overnight in a moist chamber at 4 °C. After rinsing in PBS, the sections were then incubated with secondary gold-conjugated antibodies in a moist chamber at room temperature for 80 min. For double labeling, sequential incubation in solutions of different primary antibodies followed by incubation in a mixture of appropriate secondary antibodies was used. The sections were contrasted with uranyl acetate. The control grids were incubated in a buffer without primary antibodies and then in gold-conjugated secondary antibodies under the same conditions as described.

#### Counting of Gold Labels and Statistical Analysis

Labels were counted in 1 μm^2^ random squares of ultrathin sections (Supplementary Figure S1). The intensity of labeling was compared between the following GV areas: NP—nucleoplasmic regions located far from the karyosphere-containing nucleolar assemblage; NPN—areas of the nucleoplasm inside the karyosphere-containing nucleolar assemblage but away from the chromosomes; NPC—the karyosphere region, i.e., nucleoplasmic areas in the close vicinity of the chromosomes; and St—areas of the nucleoplasm occupied by the strands. When comparing the intensity of labeling between these GV areas, an analysis of variance (one-way ANOVA test) with multiple pairwise comparisons according to the Tukey procedure was performed using GraphPad Prism 9.3.1 (GraphPad Software). Differences were considered significant at *p* < 0.05.

### 2.7. Proteomic Analysis

Samples were subjected to denaturation in 8M of urea, reduction with dithiothreitol, alkylation with 2-iodoacetamide, and digestion with trypsin. All the digests were desalted and concentrated in Strata-X 30-mg solid-phase extraction tubes (Phenomenex, Torrance, CA, USA). Resuspended peptides were separated with a Chromolith CapRod RP-18e reversed-phase column (0.1 mm × 150 mm, Merck, Darmstadt, Germany) on a nano-LC system (Eksigent NanoLC Ultra 2D+ system, SCIEX, Darmstadt, Germany). A total peptide amount of 700 ng was loaded and separated using a linear gradient of acetonitrile at a flow rate of 600 nL/min. The effluent from the column was mixed with a matrix solution containing two calibration standards—the fragment 2–9 of bradykinin and the fragment 18–39 of the human adrenocorticotropic hormone—at a flow rate of 2.4 μL/min. A micro-fraction collector was used to deposit 1 mm spots every 2 s, and a total of 1408 spots were collected in a 44 × 32 array for each nano-LC run.

The fractionated samples were analyzed with a TOF/TOF 5800 System (SCIEX) instrument operated in the positive ion mode. MS data were acquired at 2800 laser intensity with 1000 laser shots/spectrum (250 laser shots/sub-spectrum), and MS/MS data were acquired at 3700 laser intensity with a DynamicExit algorithm and a high spectral quality threshold or a maximum of 1500 laser shots/spectrum (250 laser shots/sub-spectrum). Up to 30 top precursors with S/N > 30 in the mass range 750–3500 Da were selected from each spot for MS/MS analysis.

The Protein Pilot 5.0.1 software (SCIEX, Darmstadt, Germany) with the Paragon algorithm in thorough mode was used for the MS/MS spectra search against the *R. temporaria* genome-derived protein database downloaded from the NCBI (assembly accession GCF_905171775.1). We accepted identifications of proteins that passed the 1% global FDR threshold. Each protein needed to be detected at least across three of six biological replicates to be included in the final list of accepted identifications. A protein abundance index (PAI) was used as a measure of protein abundance for graphical representation. The PAI was defined as the total number of MS/MS fragments identified per protein, normalized by sequence length. Functional annotation and gene ontology (GO) enrichment were performed using the STRING database [34], and the data were visualized with Cytoscape [35].

## 3. Results

The latest vitellogenic oocytes of *R. temporaria* ready for ovulation were used in this study. In such oocytes, the most noticeable GV structure is a giant (more than 150–300 μm) conglomerate of amplified nucleoli (Figure 1a), also referred to as the nucleolar cloud or assemblage. Condensed post-lampbrush chromosomes are tangled into a compact unit—the karyosphere (Figure 1b)—located in the center of this nucleolar assemblage.

When manually isolated from the GV, the superstructural karyosphere-containing complex is easily visible under a binocular microscope and retains its integrity for a short time after isolation. It allowed us to collect these complexes for proteomic analysis (Section 3.1). However, it should be emphasized that it is impossible in principle to solely isolate the karyosphere; therefore, preparations of entire nucleolar complexes containing the karyosphere inside are mentioned here as karyosphere preparations.

### 3.1. Proteomic Analysis of the Karyosphere

**Figure 2:**
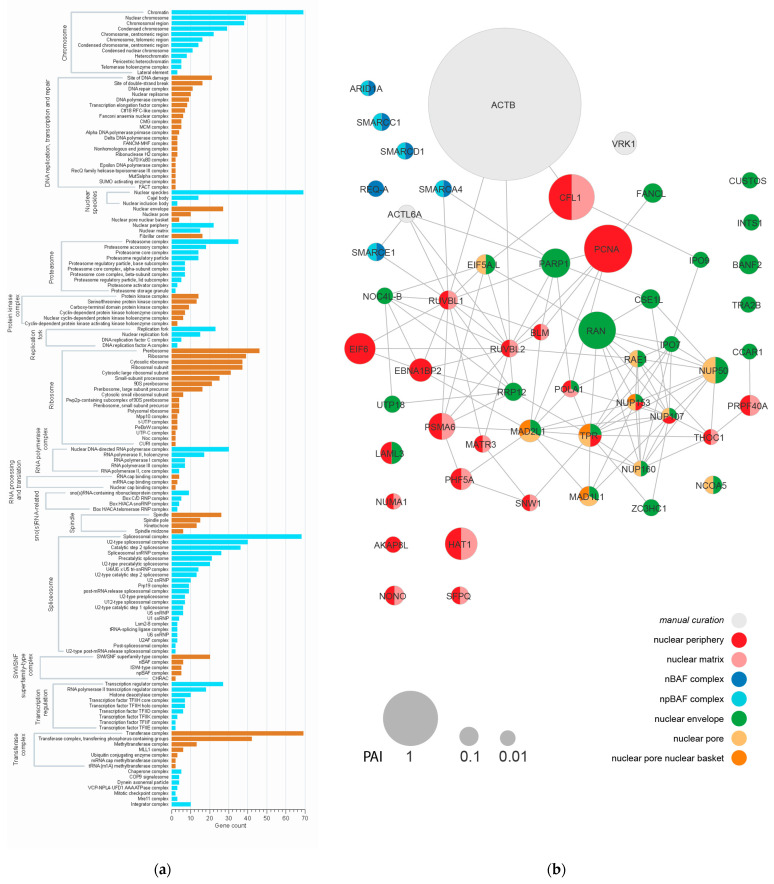
Proteomic analysis of the karyosphere region shows major protein clusters and reveals some nuclear envelope proteins. (**a**) The GO cellular component (CC) terms significantly enriched in the karyosphere proteome show major structural and functional protein clusters. (**b**) Protein interaction network predicted using STRING for the set of proteins related to the “nuclear periphery”, “nuclear matrix”, “nuclear envelope”, “nuclear pore”, and “BAF complex” GO CC terms. The node size corresponds to the protein abundance index (PAI); the node color represents a particular GO CC term.

We performed an LC-MALDI shotgun proteomic study of karyosphere-containing nucleolar complexes isolated from *R. temporaria* late vitellogenic oocytes, expecting to identify some NE proteins in the putative KC. A total of 778 protein groups were identified (Supplementary Table S2). A GO enrichment analysis of cellular components (CC) revealed 15 major structural and functional protein clusters (Figure 2a). As expected, many identified proteins were related to the karyosphere itself and pre-mRNA processing, namely, those associated with chromatin, spliceosomes, and nuclear speckles. Some proteins were specifically related to condensed nuclear chromosomes, a fact which apparently reflects the condensed state of the bivalents assembled into the karyosphere. As also expected, abundant were also the proteins associated with the nucleoli and related to ribosome biogenesis, since amplified nucleoli represent the bulk of the karyosphere-containing complexes which were used for the proteomic analysis.

Among the non-chromosomal and non-nucleolar proteins, according to the PAI, the most abundant protein was actin, especially ACTNB (Figure 2b). Moreover, the proteins of transferase complexes and proteasomes were also noticeable among the identified proteins.

In the context of our study, most intriguing was the enrichment of the karyosphere samples in some NE-related proteins, although NEs had been carefully removed during the preparation of the karyosphere complexes. In particular, these include proteins belonging to the following groups: “nuclear periphery”, “nuclear matrix”, “nuclear envelope”, “nuclear pore”, and “BAF complex” (Figure 2a). Further STRING analyses of 54 proteins related to these terms revealed a network with 113 edges and an average node degree of 4.19 (Figure 2b).

The most connected were RUVBL1, RUVBL2, TRP (14 edges each), and NUP107 (13 edges), but all these proteins are beyond the scope of this study. More intriguing was the identification of proteins associated with the NE and/or the “nuclear matrix” in the karyosphere preparations used in the proteomic analysis. This allowed us to continue our search for *R. temporaria* KC using the ultrastructural localization analysis of some representative proteins.

### 3.2. Morphology of Extrachromosomal Structures in Frog GV

Since proteomic analysis, during which proteins related to the NE and the “nuclear matrix” were identified in the karyosphere preparations, does not allow one to localize the proteins of interest in any special compartment of the GV, we performed an electron microscopic study. We paid special attention to the extrachromosomal structures—strands and *annuli*—that are considered the main candidates for the role of KC elements [19]. The presence of these strands and *annuli* in the *R. temporaria* GV was also confirmed in our previous work [36].

**Figure 3:**
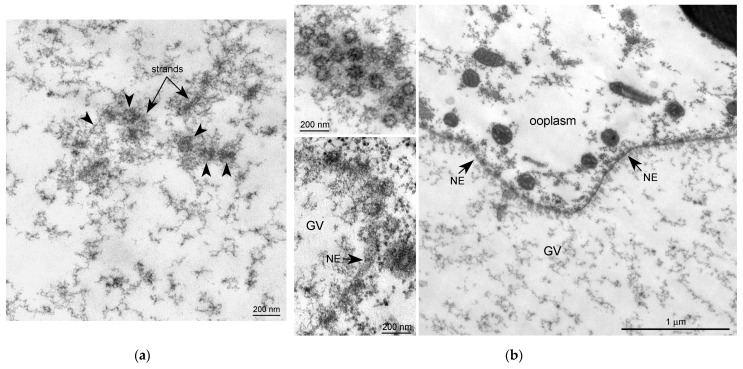
Ultrastructural morphology of the *annuli* and nuclear pore complexes in the *Rana temporaria* germinal vesicle. (**a**) A fragment of the strand material; the arrowheads show the *annuli*. (**b**) Nuclear envelope (NE, arrows) of the *R. temporaria* germinal vesicle (GV); the insets on the right show the nuclear pore complexes cut at different angles. Note a morphological similarity between the nuclear pore complexes and the *annuli*.

**Figure 4:**
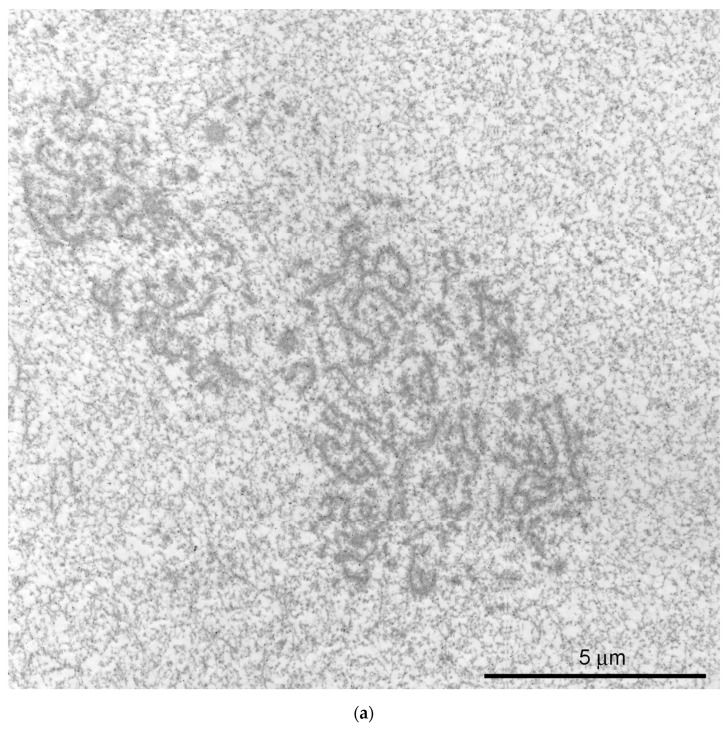

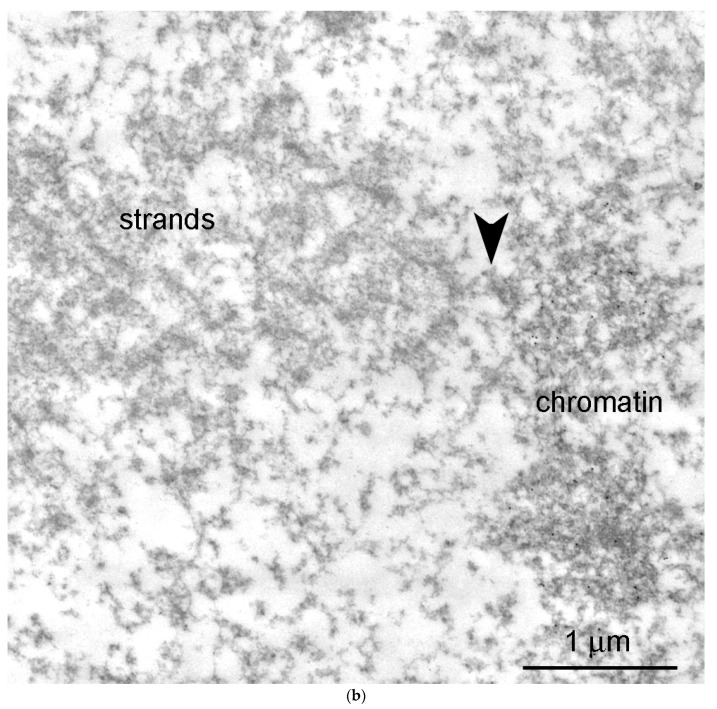
Extrachromosomal strands in the *R. temporaria* germinal vesicle: (**a**) General view of the nucleoplasmic region occupied by the strands, as viewed using immunogold labeling to find the actin; and (**b**) strands located in close proximity to chromatin, demonstrating a physical association with it (arrowheads), as viewed using DNA labeling (10 nm gold particles) to distinguish the chromatin from the extrachromosomal structures.

At the ultrastructural level, some fragments of an extrachromosomal filamentous material were observed at a noticeable distance from the karyosphere. This material—the strands—did not form a continuous shell/layer/capsule around the chromosomes assembled in the karyosphere, being poorly distinguishable from masses of condensed chromatin under a light microscope (Figure 1a). These filamentous strands, 40–50 nm in thickness, bind the 65–75 nm structures—the *annuli* [19]—and form irregular-meshwork areas in the GV. Noticeably, the *annuli* are morphologically very similar to the nuclear pore complexes (Figure 3). The areas of the strand meshwork can occupy fairly spacious regions of the nucleoplasm (Figure 4a). Some strands protrude into the karyosphere region and can sometimes be observed in close proximity to the chromosomes (Figure 4b).

### 3.3. Nuclear Actin, Lamin, and the Extrachromosomal Strands

**Figure 5:**
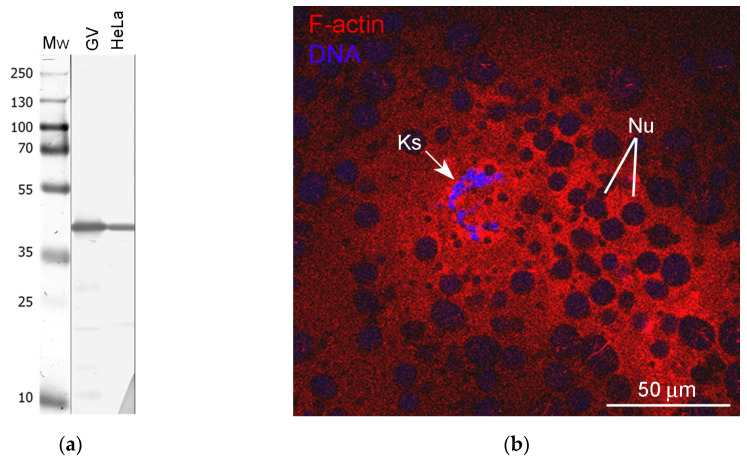
Detection of nuclear actin in the *R. temporaria* germinal vesicle: (**a**) Western blotting of GV and HeLa cell extracts with antibodies against the N-terminus of actin, 10% SDS-PAGE; (**b**) Karyosphere (Ks)-containing fragment of the germinal vesicle after rhodamine-phalloidin staining, demonstrating a significant concentration of F-actin in this region. The unstained “holes” of varying sizes represent numerous amplified nucleoli (Nu) assembled into a large nucleolar assemblage containing the karyosphere.

Here, we tested whether structural proteins such as actin and lamins could be components of any extrachromosomal entities in the *R. temporaria* GV. Since it has long been known that amphibian GVs contain enormous amounts of nuclear actin, about a third of which is in a polymerized form [23], and actin filaments are very abundant in the *R. temporaria* GV [24], we first wished to test whether the peculiar extrachromosomal strands mentioned above could represent bundles of F-actin filaments. To reveal actin as a whole, including in the ultrathin sections, we used an antibody against the N-terminus. The specificity of this antibody was demonstrated in blots of extracts of both *R. temporaria* GVs and HeLa cells (Figure 5a). Phalloidin staining revealed that F-actin clearly concentrates in the chromosome-containing area of the GV and around the karyosphere (Figure 5b), which confirms previous observations [37]. Notably, this staining pattern seems to suggest the presence of a prominent KC, but our further studies cast doubt on this (see below).

**Figure 6:**
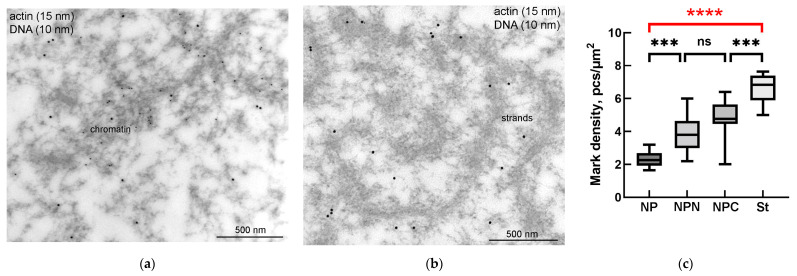
Actin distribution in the *R. temporaria* germinal vesicle: (**a**) a fragment of condensed chromatin in the karyosphere, as viewed using immunoelectron microscopy with antibodies against dsDNA (smaller particles of 10 nm) and the N-terminus of actin (larger particles pf 15 nm); (**b**) fragment of an extrachromosomal strand after the same labeling (no DNA labeling is observed in this case, since the strands are extrachromosomal structures); (**c**) labeling density of different parts of the germinal vesicle (for abbreviations NP/NPN/NPC/St used here and further, see Materials and Methods (Section 2.6). Differences are indicated as follows: ***, *p* < 0.001; ****, *p* < 0.0001; ns, not significant, *p* > 0.05. Significant differences between the germinal vesicle regions—the most considerable for the study—are hereinafter marked in red.

To prove whether the *R. temporaria* GV indeed contains an actin-rich KC, we performed a comprehensive ultrastructural study. In the ultrathin sections after immunogold labeling, actin labels were found scattered throughout the nucleoplasm, including in the karyosphere region (Figure 6a) and in regions occupied by the strands (Figure 6b). To compare the concentration of labels in different regions of the GV, we counted the number of gold particles as described in the Materials and Methods section. Although the strands themselves contain little or no actin (Figure 6b), as was previously shown [36], the concentration of labels in the areas occupied by the strands was ~3 times higher than in the GV areas located at a considerable distance from the karyosphere (Figure 6c). In this case, an increase in the concentration of actin labels was detected from the periphery of the GV to its center (Figure 6c), as previously assumed based on the results of non-quantitative electron microscopic observations [24].

**Figure 7:**
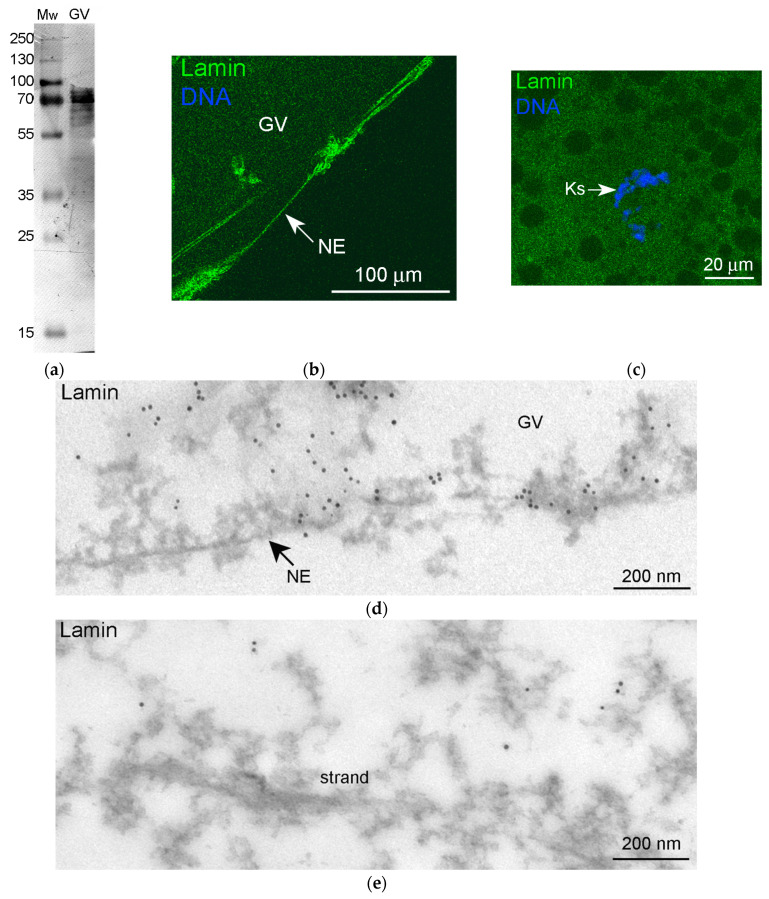
The revealing of a lamin protein in the *R. temporaria* germinal vesicle (GV) with a lamin B antibody: (**a**) Western blot analysis, 10% SDS-PAGE; (**b**,**c**) immunofluorescent staining of the GV periphery, including the nuclear envelope (NE) (**b**) and the karyosphere (Ks)-containing area (**c**); (**d**,**e**) immunogold labeling of the GV periphery (**d**) and area of the strands (**e**). Note that the strand is completely unlabeled.

Apart from nuclear actin, lamins of the B type—perhaps amphibian oocyte-specific lamin B3, called LIII [38]—are abundant in the karyosphere-containing part of the *R. temporaria* GV [28]. To test whether lamin B protein is included in the strands, we immunolabeled *R. temporaria* GVs with an anti-lamin B antibody that reacts with GV extracts in Western blots (Figure 7a). As expected, we detected a bright staining of the NE (Figure 7b). However, only a weak and blurred staining of the karyosphere region was observed (Figure 7c). At the ultrastructural level, this antibody also marked the nuclear lamina (Figure 7d). In addition, the labels were also scattered throughout the nucleoplasm, but the strands themselves remained almost completely unlabeled (Figure 7e).

### 3.4. Nucleoporins and Annuli

**Figure 8:**
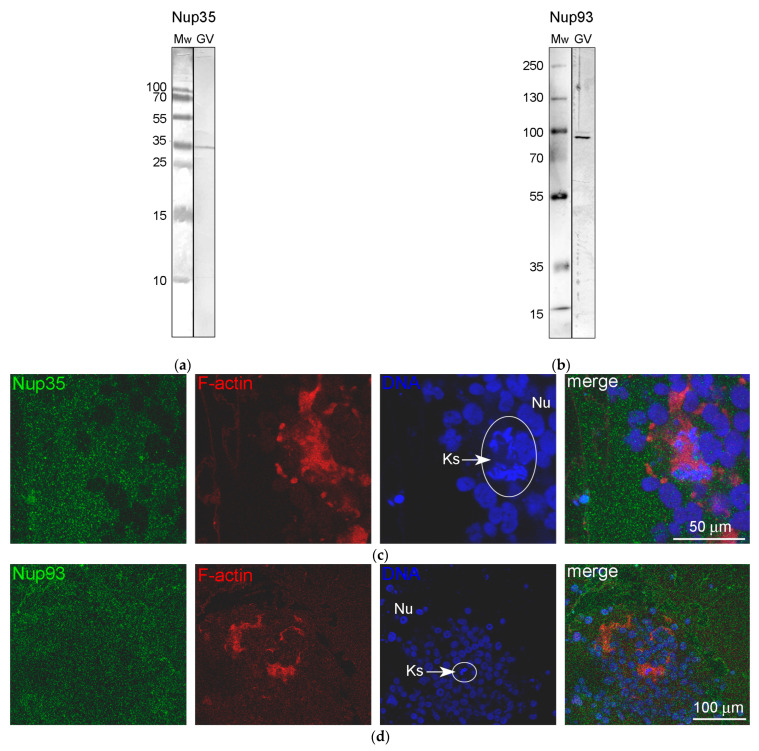
Detection of nucleoporins in the *R. temporaria* germinal vesicle (GV): (**a**,**b**) Western blot analysis with Nup35 (**a**) and Nup93 (**b**) antibodies, 15% and 8% SDS-PAGE, respectively; and (**c**,**d**) localization of Nup35 (**c**) and Nup93 (**d**) in the karyosphere regions (Ks, encircled) using immunostaining. F-actin is counterstained with rhodamine-phalloidin (red), while the DNA (karyosphere) is counterstained with DAPI (blue).

**Figure 9:**
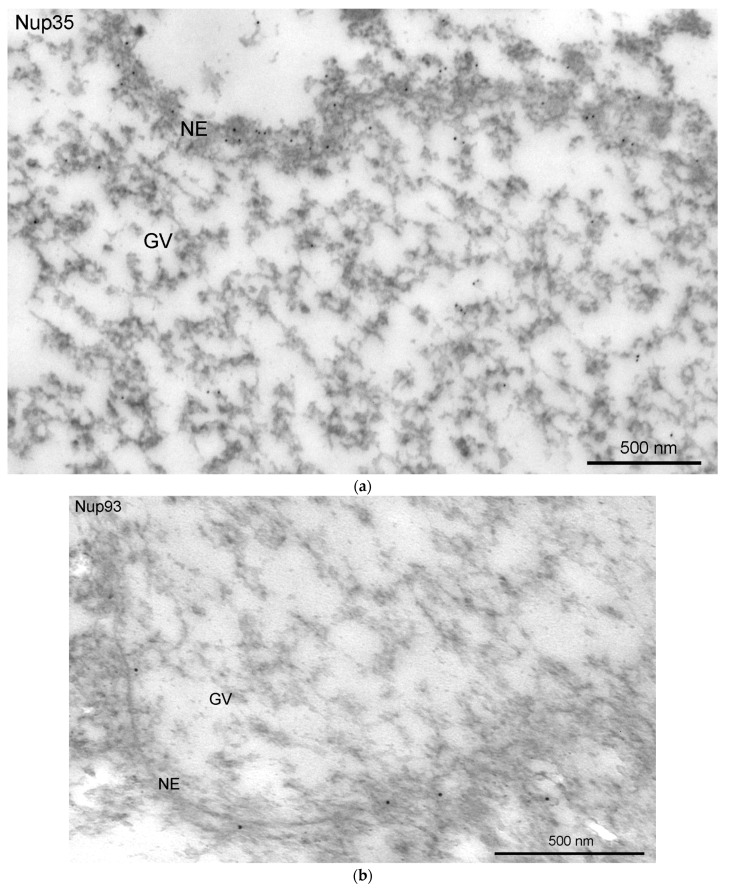

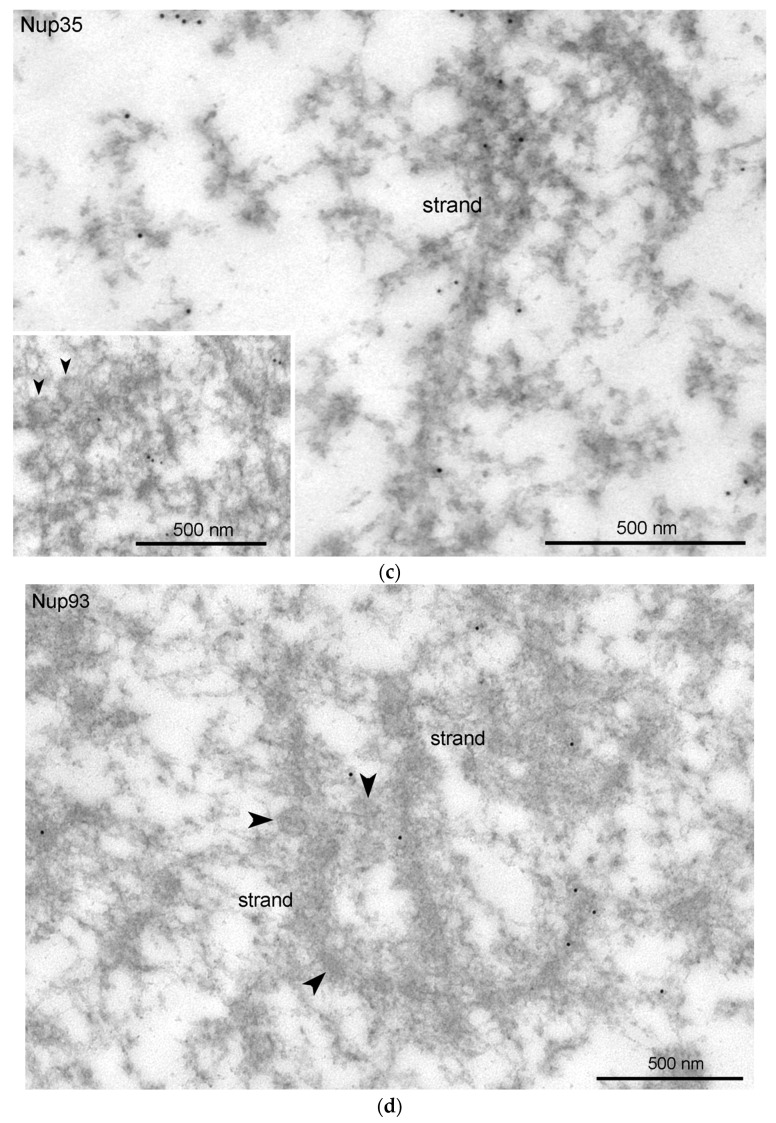
Detection of nucleoporins in the *R. temporaria* germinal vesicle at the ultrastructural level: immunoelectron localization of Nup35 (**a**,**c**) and Nup93 (**b**,**d**) in the nuclear envelope (NE) (**a**,**b**) and in strands and *annuli* (**c**,**d**). Some representative *annuli* are pointed at with arrowheads.

We used antibodies to two representative nucleoporins of the inner ring of the NE pore complexes, namely Nup35 and Nup93 [39]. These antibodies showed a specific reaction with the proteins of *R. temporaria* GV extracts in Western blots (Figure 8a,b). At the fluorescence level, we did not reveal staining of any specific structures in the inner regions of the GV, including the karyosphere region (Figure 8c,d). However, the antibodies to Nup35 and Nup93 labeled the NE, demonstrating that this labeling is specific (Figure 9a,b). At a distance from the NE, the nucleoplasm demonstrated diffuse labeling, including in areas near some strands (Figure 9c,d). Noticeably, the so-called *annuli*—characteristic structures of the *R. temporaria* GV—apparently do not contain neither Nup35 nor Nup93 (Figure 9c,d), despite their clear morphological resemblance to the nuclear pore complexes (Figure 3).

### 3.5. Nuclear Envelope-Related Proteins and the “Karyosphere Capsule”

Although the vast majority of intranuclear structures, including the KC [7], are non-membrane entities, in search of any possible analogies between the putative KC and the NE, we decided to check whether the extrachromosomal strands—the characteristic structures of the central region of *R. temporaria* GVs, in which the karyosphere is located—may be associated with proteins that ensure known chromatin–NE interactions in somatic cells [4]. Among these proteins, we paid special attention to BANF1/BAF, which plays a key role in the interaction of chromatin with LEMD proteins, integral proteins of the inner nuclear membrane [40].

**Figure 10:**
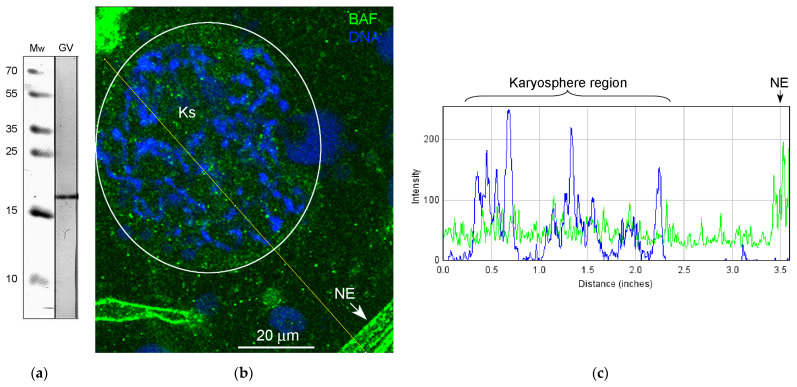
Detection of BAF in the *R. temporaria* germinal vesicle: (**a**) Western blot analysis, 15% SDS-PAGE; (**b**) a fragment of the germinal vesicle after immunostaining with the BAF antibody (the karyosphere (Ks) region is encircled, and the DNA is counterstained with DAPI), NE, nuclear envelope (it often forms artifactual folds in squashed GV preparations); and the (**c**) RGB plot of the selected profile.

**Figure 11:**
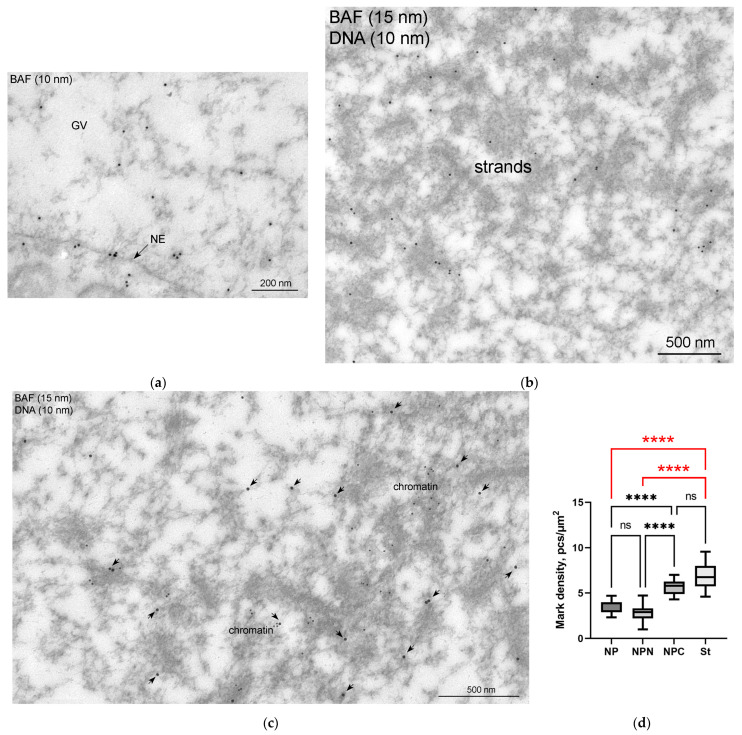
Ultrastructural localization of BAF in the *R. temporaria* germinal vesicle: (**a**) a peripheral part of the germinal vesicle (GV), demonstrating the labeling of the nuclear envelope (NE), as well as the fact that the labels are also scattered in the nucleoplasm; (**b**,**c**) double immunogold labeling with BAF and DNA antibodies (15 and 10 nm gold particles, respectively), after which the strands remain unlabeled (**b**), whereas the BAF labels (enhanced using arrows) are located in the vicinity of condensed chromatin (**c**); (**d**) counting of the labeling density of different parts of the GV, indicating a concentration of BAF in the karyosphere regions of the GV. Differences are indicated as follows: ****, *p* < 0.0001; ns, not significant, *p* > 0.05; the most essential differences in the study context are shown in red.

The presence of BAF in the *R. temporaria* GV was confirmed using Western blotting (Figure 10a). Immunofluorescence microscopy demonstrated a fairly uniform distribution of BAF throughout the GV, including in the karyosphere region (Figure 10b,c). The exception is the NE, fragments of which are stained intensely (Figure 10b,c), a matter which is also evident at the ultrastructural level (Figure 11a). In the ultrathin sections, the labels indicative for the localization of BAF were found scattered throughout the GV, including near the strands (Figure 11b) and chromatin (Figure 11c), but no specific labeling of the strands themselves was detected. However, the density of BAF labeling was found to be higher in regions of both the karyosphere and strands compared to regions of the nucleoplasm away from the karyosphere (Figure 11d), despite the fact that this is not obvious when BAF is visualized using fluorescence microscopy.

**Figure 12:**
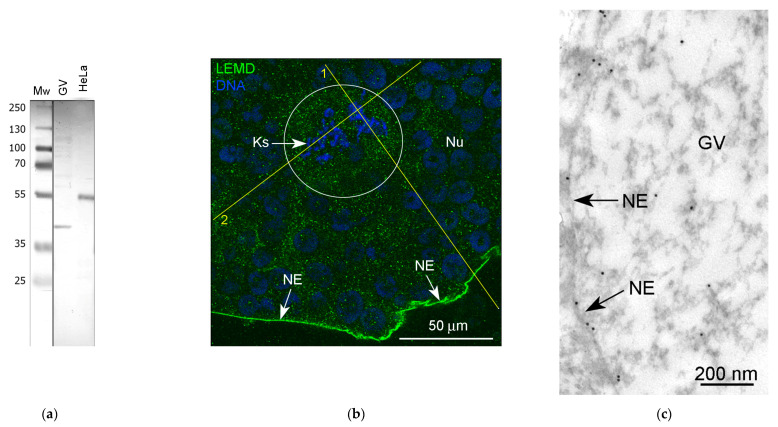

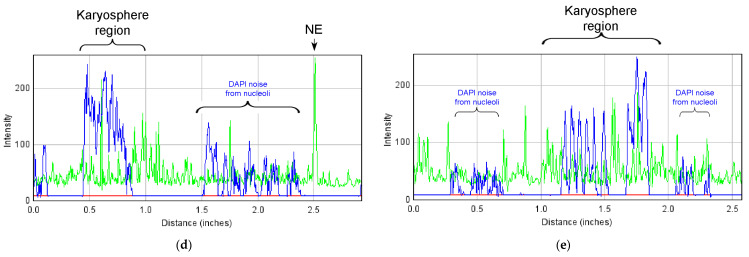
Detection of LEMD protein in the *R. temporaria* germinal vesicle (GV): (**a**) Western blot analysis, 10% SDS-PAGE; (**b**) fragment of GV immunostained with the LEMD2 antibody, in which the karyosphere (Ks) region is encircled; (**c**) immunogold labeling of the nuclear envelope (NE) with the LEMD2 antibody; (**d**,**e**) RGB profiles corresponding to the lines 1 (**d**) and 2 (**e**) shown in (**b**). Note that, in addition to the fairly diffuse staining of the nucleoplasm, including the karyosphere region, this antibody provides a strong staining of the NE.

Taking into account that the BAF is one of the key interactors of the LEMD (Lap2-Emerin-Man1-domain) family of inner nuclear membrane proteins (for a review, see [4]), we would like to recognize a LEMD protein in the *R. tempotatia* GV. In the Western blots, for which we had available a commercial antibody to the recombinant protein corresponding to human LEMD2, a conserved protein from yeast to humans [41], a ~55 kDa protein in the HeLa cell extracts was recognized, as expected. However, the size of the recognized band in the *R. temporaria* GV extracts was ~40 kDa (Figure 12a). Nevertheless, we considered it possible to use this antibody in our immunocytochemical experiments on frog GVs, since no other bands were detected in the blots and this antibody brightly stains the NE using both fluorescence (Figure 12b) and immunogold labeling/electron microscopy (Figure 12c).

In addition to the intense staining of the nuclear envelope, a moderate and rather even staining of the nucleoplasm was also observed, including in the karyosphere region (Figure 12b). The RGB profiles (Figure 12d,e) confirm a rather diffuse LEMD2 distribution in the karyosphere region of the GV.

**Figure 13:**
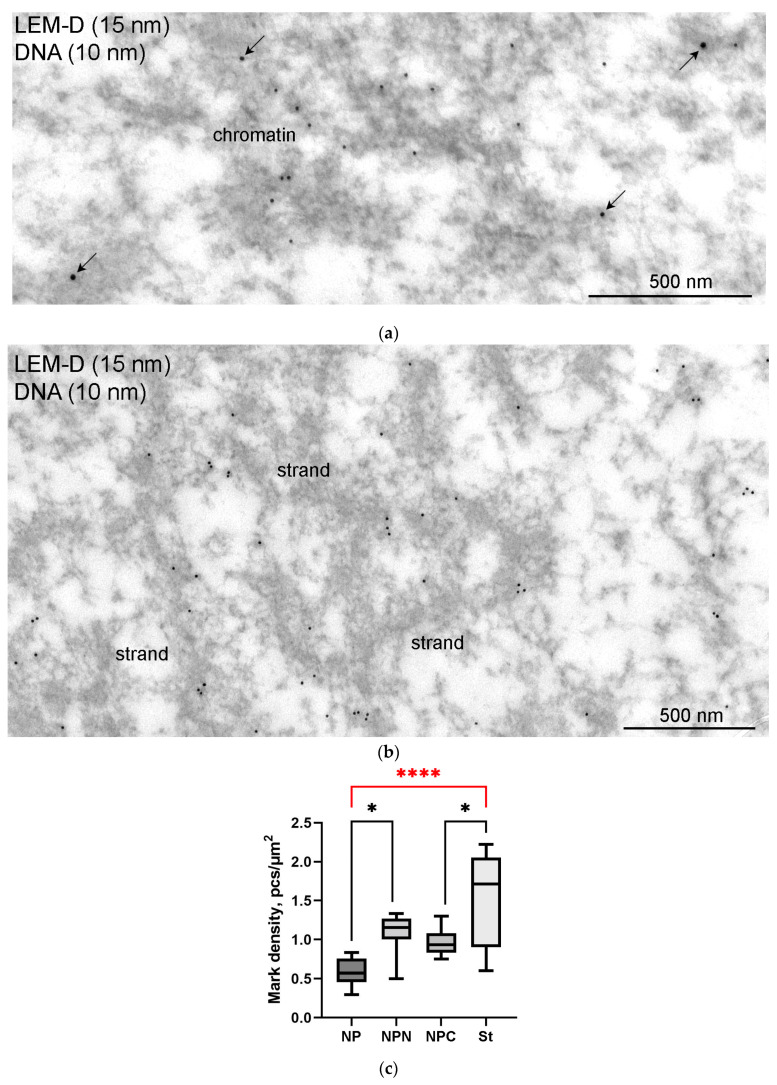
Double immunogold labeling of chromatin (**a**) and the strands (**b**) with antibodies to dsDNA (10 nm gold particles) and LEMD2 (15 nm gold particles, some additionally enhanced using arrows); (**c**) counting of the labeling density of different GV parts. Despite the fact that LEMD2 is distributed throughout the GV, it concentrates in the strand-containing parts of the nucleoplasm (****, *p* < 0.0001); differences between other parts of the GV are not so significant (*, *p* < 0.05).

At the ultrastructural level, LEMD labels were also observed scattered throughout the nucleoplasm. Some labels were clearly seen in the close vicinity of chromatin (Figure 13a) and extrachromosomal strands (Figure 13b). The counting of label density revealed a little difference between different regions of the GV, except for the strand regions, where the concentration of labels was more than 2.5 times higher than in the nucleoplasmic areas far from the karyosphere (Figure 13c).

### 3.6. SMC1 Is a Component of the Strands

Finally, we studied a possible association of the SMC protein with the frog’s karyosphere, bearing in mind the following considerations: (i) the karyosphere is a complex structure that forms at the diplotene stage of meiotic prophase due to the assemblage of condensed chromosomes into a rather compact structure [8]; (ii) chromosome architecture depends largely on the cohesin complex [42], which organizes chromatin into three-dimensional structures [43]; (iii) structural maintenance of chromosomes (SMC) proteins—the key components of the SMC complex [44]—play a role in karyosome formation, at least in *Drosophila* [45]; and (iv) the SMC complex maintains chromatid cohesion for a long time in meiotic cells [46], until the resolution of chiasmata during the first meiotic division [47].

**Figure 14:**
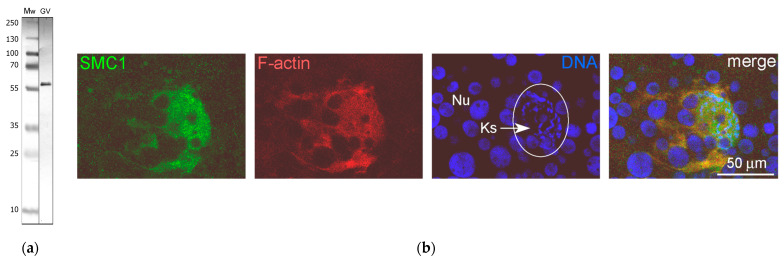
Detection of the SMC1 protein in the *R. temporaria* germinal vesicle (GV): (**a**) Western blot analysis, 10% SDS-PAGE; (**b**) karyosphere-containing part of the GV after staining with the anti-SMC1 antibody SMC1L1 (green), rhodamine-phalloidin (red), and DAPI (blue). The chromosomes assembled into the karyosphere (Ks) are indicated using an arrow. Nu, amplified nucleoli.

**Figure 15:**
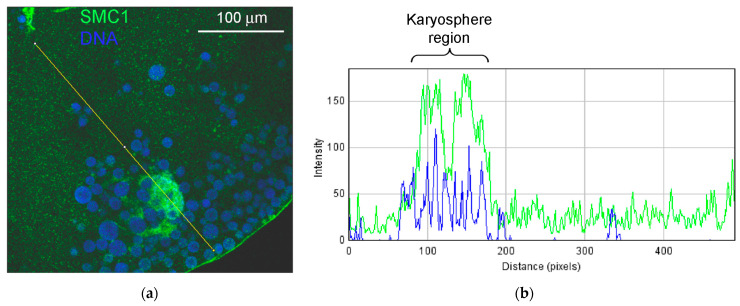
Distribution of the SMC1 in the *R. temporaria* germinal vesicle: (**a**) fragment of the germinal vesicle with the karyosphere-containing region, and (**b**) the RGB plot of the selected profile. Note the strong localization of the SMC1 (green) in the karyosphere region; DNA is stained with DAPI (blue).

**Figure 16:**
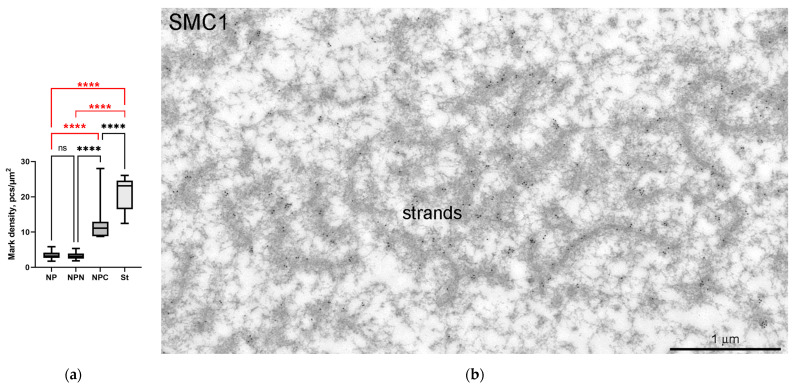

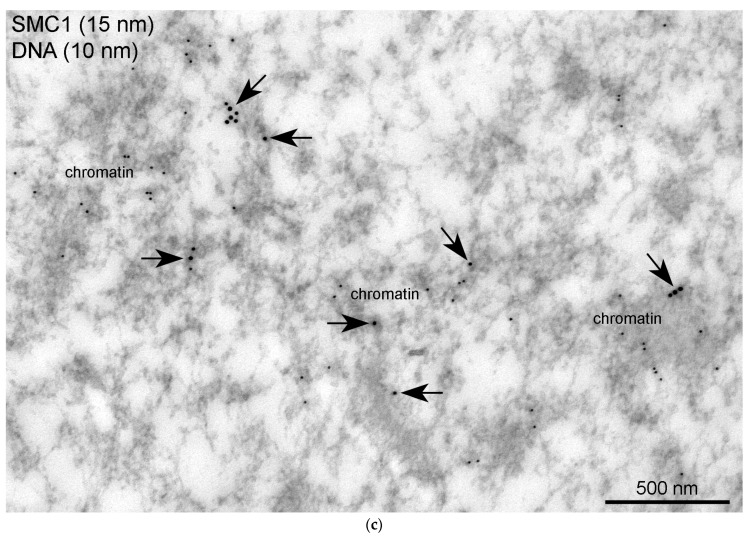
Localization of the SMC1 in the *R. temporaria* germinal vesicle at the ultrastructural level: (**a**) counting of the labeling density in different parts of the germinal vesicle after immunogold labeling with an anti-SMC1L1 antibody, showing that SMC1 is concentrated in the karyosphere-containing part of the GV, including in the strands (****, *p* < 0.0001; ns, not significant, *p* > 0.05); (**b**) electron microscopy image demonstrating the clear localization of the SMC1 in the extrachromosomal strands; (**c**) fragment of condensed chromatin of the *R. temporaria* karyosphere after double labeling with antibodies against SMC1A (15 nm gold particles, arrows) and dsDNA (10 nm gold particles).

The monoclonal antibody SMC1L1 gave a single band after Western blotting with *R. temporaria* GV proteins (Figure 14a). When applied in an immunocytochemistry assay, this antibody revealed a protein localized predominantly in the karyosphere region of the GV (Figure 14b), while areas of the nucleoplasm distant from the karyosphere showed only weak SMC1 staining (Figure 15). The localization pattern of the SMC1—or rather its frog orthologue—was confirmed at the ultrastructural level (Figure 16), including a gold particle count approach (Figure 16a). Importantly, the extrachromosomal strands (Figure 16b) were clearly labeled with anti-SMC1 antibodies. In addition, SMC1 was found to colocalize with the karyosphere’s condensed chromatin, as expected (Figure 16c). Therefore, unlike other the proteins studied—the increased concentration of which is observed in the strand regions, although the strands contain little to no amount of these proteins—SMC1 is predominantly localized in the strands themselves.

## 4. Discussion

### 4.1. What Does the Word “Capsule” Mean?

There are two terms to describe similar GV structures—karyosphere and karyosome. Although these terms are close in meaning, even the American entomologist Maulsby Blackman—the discoverer of the karyosphere—wrote the following: “I have limited the term karyosome to structures … which are apparently composed exclusively of chromatin. The karyosphere is much more highly organized… It is in fact a miniature nucleus.” [48]. For example, a typical karyosome develops in the Drosophila GV, which is a rather simple tangle of chromosomes [49]. On the contrary, the formal resemblance of the karyosphere—a more complex structure—to a “nucleus in the nucleus” (cf. German Innenkern—Vejdovský, 1911–1912, cited by [7]) had been emphasized in many works (for the historical information, see Table 1 in the paper [7]), especially due to the discovery of the karyosphere capsule (KC) in the GV of the common frog R. temporaria [18]. The R. temporaria KC has long been considered to be a specialized element of the nuclear matrix [7], although a decade ago the concept of the nuclear matrix was seriously criticized [50].

Despite the long history of the KC and the well-established ideas about its existence as a special extrachromosomal compartment which further separates the chromosomes assembled in the karyosphere from the rest of the nucleoplasm at the end of the growth period of *R. temporaria* oocytes [18,19,29,51], the data obtained in the previous [36] and present studies cast doubt on this issue, at least for this frog species.

In a general sense and in its simplest form, a capsule (Latin *capsula*) is a small box or container. It is in this sense that this word (German *Kapsel*) was introduced to describe a fibrous substance (German *faserigen Substanz*) visible around the chromosomes of the *R. temporaria* GV after Heidenhain’s iron hematoxylin staining [18]. Later, already at the ultrastructural level, specific structures—the strands and *annuli*—were discovered in the karyosphere-containing region of the *R. temporaria* GV, which led to the idea that these structures were elements of the Wagner capsule [19]. Although we have re-discovered these strands and *annuli* ([36], and the present work), the extrachromosomal material of the *R. temporaria* GV does not form a prominent continuous layer/shell around the karyosphere, sometimes occupying voluminous but still limited areas of the nucleoplasm.

At the same time, an enormous KC actually exists in the GV of some insects, such as in the neuropterans [20,26] and in some coleopterans [25,27] but not in another coleopteran, *Tenebrio molitor* [52], or in the fruit fly *Drosophila melanogaster* [49], with their capsule-less karyosomes (for details, see [8]). When the KC develops in insect GVs, it contains a lot of F-actin, which allows it to be considered a signature component of the KC [8]. Although insect KC is not always a closed compartment [26], it is a solid-like and extensively developed complex structure, as viewed at the ultrastructural level. The most prominent, actin-containing electron-dense part of the KC was referred to as a “shell of the capsule” [27]. Nothing similar to such a “shell” is observed in the *R. temporaria* GV.

### 4.2. Extrachromosomal Strands Do Not Contain Structural Proteins Actin and Lamin B

Our proteomic analysis of karyosphere complexes showed that they contain a large amount of β-actin. This is not surprising, since the actin pool in the cell nucleus is predominantly limited to only one isoform—β-actin [53]. It is also known that, unlike any somatic cell, amphibian GV contains a huge amount of nuclear actin, which accounts for 6% of all GV proteins at a concentration of ~2 mg/mL [54]. This is because exportin-6—a specific nuclear actin export factor—is not expressed in the GV [55]. Moreover, in the *Xenopus laevis* GV, over 37% of the total nuclear actin is F-actin [56], which can be visualized using routine phalloidin staining [57], as opposed to the somatic nucleus. However, even in the somatic nucleus, the F-actin network is highly dynamic and contributes significantly to the spatiotemporal organization of the genome and nuclear architecture [58].

The presence of a dense network of actin filaments in amphibian GV was confirmed using electron microscopy for both *X. laevis* [59] and *R. temporaria* [24]. However, with such a high concentration of nuclear actin in amphibian GVs, it can instantly gel when the GV is manipulated [23]. This apparently can explain the possibility of manually isolating the *R. temporaria* karyosphere enclosed in a jelly-like “capsule” [19].

The characteristic pattern of phalloidin staining that demonstrates a high concentration of F-actin around the karyosphere, creating a clear illusion of a KC in frog oocytes [28,37], had also promoted further development of the ideas on the existence of *R. temporaria* KC as a conserved special GV compartment [8]. However, unlike the GV of a beetle *Tribolium castaneum* [27,36], in the *R. temporaria* GV we did not find actin-containing structures that could resemble a filamentous KC at the ultrastructural level. However, according to our data presented here, actin concentration in the karyosphere region of the *R. temporaria* GV is indeed significantly higher than in off-lying peripheral areas, which can explain the results of the phalloidin fluorescent staining. Only the additional use of electron microscopy helped us resolve this issue. The most important conclusion following our observations is that the special strands characteristic of *R. temporaria* GVs contain little to no actin, although they are located in the actin-rich region of the GV, which is intensely stained with phalloidin at the light microscopy level.

When actin polymerization in the GV is disrupted with latrunculin B, this leads to a collapse of nuclear structures [60]. In *R. temporaria*, this does not affect the karyosphere itself (chromosomes), but all the amplified nucleoli—which normally constitute the nucleolar assemblage located in the center of the GV—merge into a single enormous droplet [37]. It is noteworthy that the authors actually destroyed the entire nuclear F-actin network, but not some special part of it, which could be considered to be the KC. Thus, the F-actin network filling the GV, including the karyosphere region, may confer overall stability to the karyosphere-containing nucleolar complex, i.e., the most prominent structure of karyosphere-stage GVs in both anuran and caudate amphibians, see [36] for a brief historical review. According to modern concepts, it is F-actin that stabilizes nuclear ribonucleoprotein organelles, which, by their nature, are liquid droplets [61]. In our opinion, the nucleolar assemblage consisting of hundreds of amplified nucleoli—nuclear organelles which primarily exhibit a liquid-droplet behavior [62]—cannot be considered a capsule, since typical KCs are filamentous entities by definition [8,18].

In addition to nuclear actin, the other major structural protein of amphibian GV is the oocyte-specific lamin LIII/B3 [38]. Indeed, group B lamins have been detected outside the NE, including in the karyosphere region of the *R. temporaria* GV [28]. The presence of lamin proteins outside the NE has previously been reported for mouse GVs [63,64], but some details in these two reports are rather controversial and require further clarification. In the context of our work, it is more important that lamin B is not localized in specific extrachromosomal strands of frog GV ([65], and the present study). Therefore, these strands cannot be considered as KC-like elements, which, a priori, must perform a structural function and contain at least actin and/or lamin structural proteins [7,8]. Interestingly, some lamin B can be revealed in unusual biomolecular condensates found in the *R. temporaria* GV [65], the nature of which remains elusive.

### 4.3. Annuli Are Not the Autonomous Pore Complexes

Despite the strict ultrastructural similarity between the *annuli* and the nuclear pore complexes [19], these *annuli* do not contain two essential nucleoporins—Nup35 and Nup93. Outside the NE, free nucleoplasmic nucleoporins are distributed throughout the *R. temporaria* GV, including in the region containing the karyosphere. The association of a nucleoporin (Nup160) with the karyosphere—not only its presence in the nuclear periphery—was also noted in late GV oocytes of mice [63]. In frog GV, Nup35 and Nup93 did not demonstrate specific localization in any specific microstructure, including the *annuli*, with the exception of some unusual nucleoporin-containing condensates in the karyosphere region [65]. Therefore, the *annuli* do not seem to represent “autonomous pore complexes”, as previously suggested [7], although we do not exclude the presence of other nucleoporins in these unusual structures found in the GV of the common frog [19] and the marsh frog [66].

### 4.4. BAF and LEMD-Proteins in the R. temporaria Germinal Vesicle

The barrier-to-integration [nuclear] factor BAF/BANF1 is a central link between chromatin, nuclear lamina, and LEMD-proteins, integrating these nuclear elements into a single functional system [3,67]. Although still poorly understood, the functional interactions of the BAF and LEMD proteins are important for the formation of male and female gametes [4]. The role of these interactions in the formation and maintenance of the *R. temporaria* karyosphere cannot be excluded, since a concentration of BAF and LEMD proteins in the karyosphere region of the GV was found to be higher than in nucleoplasmic areas located far from the karyosphere. However, we did not find a noticeable association of these proteins with any specific GV structures.

By definition, the KC is not a membrane compartment, like the vast majority of intranuclear compartments. In this regard, the term “pseudomembranes” was proposed to describe the extrachromosomal strands observed in the GV of the common frog *R. temporaria* [7,51] and the marsh frog *Pelophylax ridibundus* [66]. Similar “pseudomembranes” have also been described in mosquito GVs [68]. The term “pseudomembrane” has apparently only historical significance and is unlikely to reflect actually existing structural–functional relationships between the karyosphere and NE proteins/structures. However, additional research is required on those species—some insects—in whose GVs the KC is actually formed.

### 4.5. SMC1 Is a Component of the Strands

The only protein that, to date, we have been able to reliably detect in the special threads of the GV of *R. temporaria* late oocytes is SMC1. SMC1 is one of the main elements of the conserved cohesin complex, also known as the structural maintenance of chromosomes (SMC) complex, which holds chromatids/chromosomes together and folds them using DNA loop extrusion [69]. According to our data, SMC1 is predominantly localized in association with the karyosphere. If the strong colocalization of SMC1 with the condensed chromatin of the karyosphere does not cause any surprise, then the localization of SMC1 in the extrachromosomal strands suggests that, in this case, we may be talking about an excess of SMC1 being involved in karyosphere formation in an earlier stage of *R. temporaria* oogenesis.

## 5. Conclusions

In our opinion, traditional ideas about the existence of the *R. temporaria* KC as a special structural compartment of the GV require revision.

Firstly, specific extrachromosomal strands contain neither actin nor lamin proteins and, therefore, do not form a structural scaffold for chromosomes assembled in the karyosphere. At the same time, actin is indeed concentrated around the karyosphere, which explains the illusion of the existence of a KC after GV staining with phalloidin. In this regard, electron microscopy has served as a good tool to help solve this problem.

It should be additionally noted that the term “pseudomembrane”, which has long been used to describe the extrachromosomal strands in the GV of various frogs [19,66] and mosquitoes [68], should be further excluded, since these strands are non-membranous entities and do not accumulate NE proteins, including LEMD2 and BAF, which provide a functional link between chromatin and the NE.

Secondly, specific *annuli* connected by the strands can hardly be unambiguously considered “autonomous pore complexes” [7], since they do not contain at least two essential nucleoporins—Nup35 and Nup93—although these nucleoporins are indeed present in the nucleoplasm. In this regard, similar *annuli* observed in mosquito GVs, which have so far only been described using conventional electron microscopy [68], also require re-examination.

**Figure 17:**
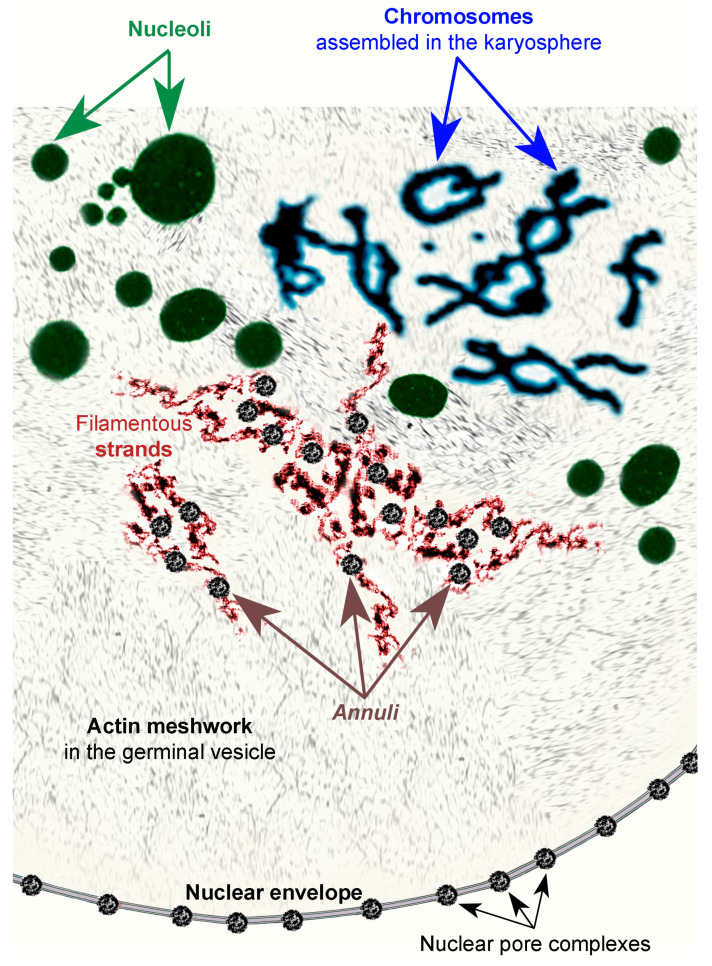
Cartoon illustrating the major nuclear structures in the germinal vesicle of *R. temporaria* late vitellogenic oocytes, excluding the histone locus bodies, the Cajal bodies, and the speckles [22], not discussed in the present paper.

In conclusion, we now believe that there is no “nucleus within the nucleus” in frog GV [7]. Therefore, the karyosphere of *R. temporaria*, in fact, is a karyosome—a simple tangle of post-lampbrush chromosomes according to our nomenclature [8]. At the same time, if someone wants (due to established traditions) to refer to the nuclear actin network and/or tangles of special filamentous strands of the *R. temporaria* GV to as a “capsule” (Figure 17), then so be it. Fundamentally, it is a question of terminology. However, one should keep in mind that well-developed fibrous KCs, the major component of which is F-actin, do exist in nature in the form of morphological structures. At the same time, they are more characteristic of the GV of some insects than of that of the common frog.

Despite the formation of a typical karyosphere (karyosome) in the GV of the common frog, our data suggest that this frog—a classic model species of developmental biology—is not a good model for analyzing the mechanisms of KC formation, since the KC is not *de facto* formed in the GV of this species.

## Data Availability

The data presented in this study are available on request from the corresponding author.

## Supplementary Materials

The following supporting information can be downloaded at https://www.mdpi.com/article/10.3390/jdb11040044/s1: Figure S1: Example of label counting procedure after immunogold labeling; Table S1: Number of animals and oocytes used in the experiments; Table S2: Proteomic analysis of karyosphere complexes isolated from the GVs of *R. temporaria* late vitellogenic oocytes.
