# Supplementary material for: Special Nuclear Structures in the Germinal Vesicle of the Common Frog with Emphasis on the So-Called Karyosphere Capsule
Source: J Dev Biol. 2023 Dec 12;11(4):44. doi: 10.3390/jdb11040044 (PMC10744300; doi:10.3390/jdb11040044)
Supplement: Supplementary file 1 [file jdb-11-00044-s001.zip › Figure_S1.pdf]

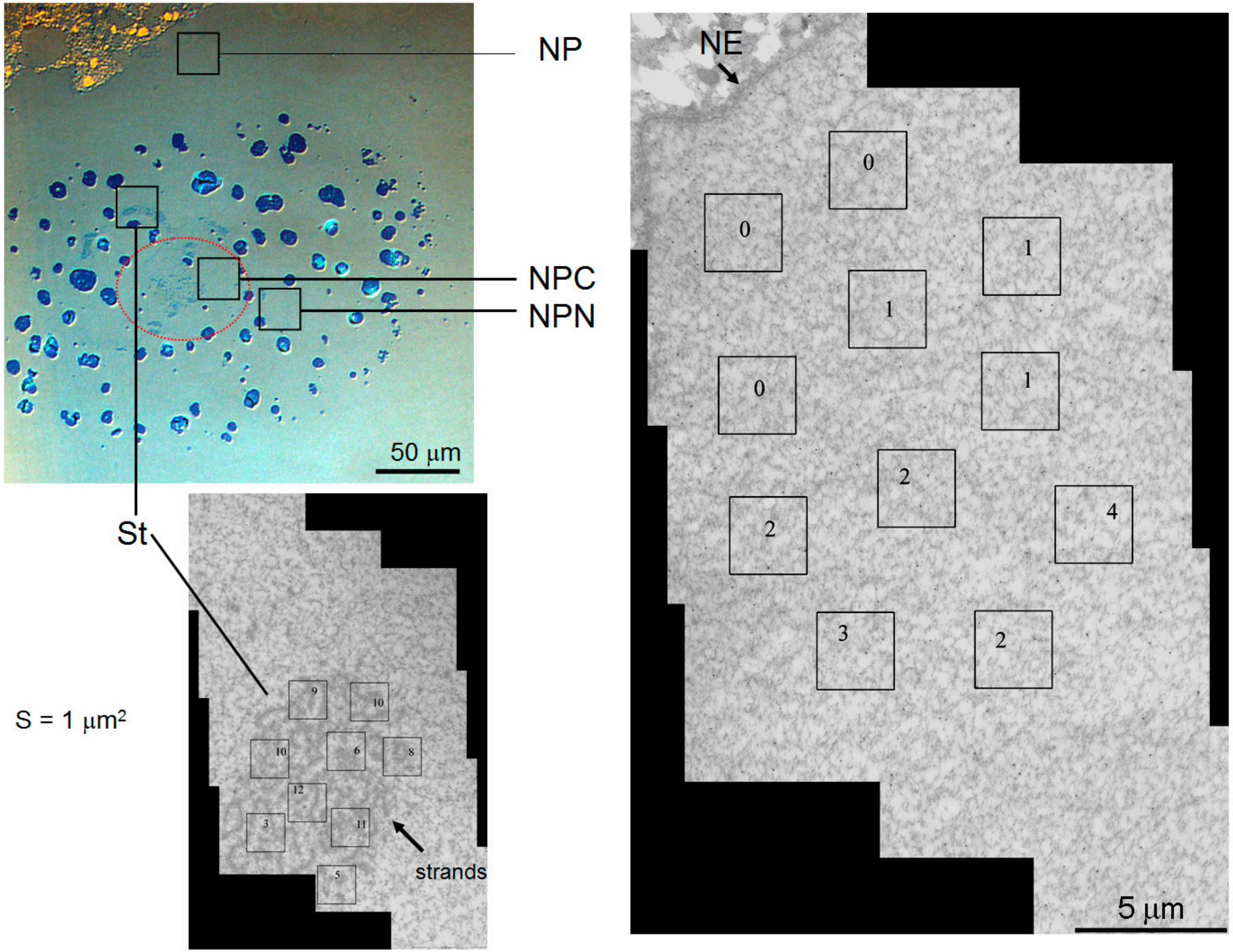

Figure 1S. Example of label counting procedure after immunogold labeling. The counting was performed in ultrathin sections in randomly selected squares of 1 µm². The numbers indicate the number of labels in the square. The compared areas of the germinal vesicle are the following: NP, peripheral regions of the nucleoplasm, i.e., located far from the nucleolar assemblage containing the karyosphere; NPN, nucleoplasm in the region of the nucleolar assemblage, but away from the chromosomes; NPC, nucleoplasm in the karyosphere region (red circle in the semithin section); St, areas of the extrachromosomal strands. NE, nuclear envelope.
