# Supplementary material for: Special Nuclear Structures in the Germinal Vesicle of the Common Frog with Emphasis on the So-Called Karyosphere Capsule
Source: J Dev Biol. 2023 Dec 12;11(4):44. doi: 10.3390/jdb11040044 (PMC10744300; doi:10.3390/jdb11040044)
Supplement: Supplementary file 1 [file jdb-11-00044-s001.zip › Table-S1_number.pdf]

Table S1. Number of animals and oocytes used in experiments.

Method	Number of Animals		Number of oocytes
	"Spring" frogs during the natural breeding season	"Autumn" and "winter" frogs after hormonal stimulation	
DAPI staining of unfixed GVs	5	6	at least 10 from each animal
Proteomic analysis	3	–	at least 30 from each animal
Immunofluorescence microscopy	5	6	at least 5 to identify each protein of interest
Conventional electron microscopy	3	–	at least 5
Immunoelectron microscopy	3	1	at least 5
Immunoblot analysis	3	–	at least 30 from each animal
